# 2‐Hydroxyisobutyrylation and Phosphorylation Crosstalk Guides Metastasis Prediction and Immunotherapy in Esophageal Squamous Cell Carcinoma

**DOI:** 10.1002/mco2.70815

**Published:** 2026-06-19

**Authors:** Junyi Li, Shujun Li, Xiaomei Yu, Qier Mu, Maowen Luo, Yuzhen Wang, Zhichao Liu, Chengwei Gu, Jiaxi Chen, Wangcheng He, Tong Yang, Yan He, Xiaoya Pei, Mao Lin, Zhigang Li, Jun Liu, Baosheng Zhao, Fan Zhang, Jinbao Liu, Bin Li, Wenwen Xu

**Affiliations:** ^1^ The Fifth Affiliated Hospital, Guangzhou Medical University, Guangzhou, China; State Key Laboratory of Metabolic Dysregulation and Prevention and Treatment of Esophageal Cancer School of Convergence Medicine Zhengzhou University Zhengzhou China; ^2^ Guangdong Provincial Key Laboratory of Protein Modification and Degradation School of Basic Medical Sciences, The Affiliated Traditional Chinese Medicine Hospital Guangzhou Medical University Guangzhou China; ^3^ The Fifth Affiliated Hospital Guangzhou Medical University Guangzhou China; ^4^ State Key Laboratory of Respiratory Disease and National Clinical Research Center for Respiratory Disease Department of Thoracic Surgery and Oncology The First Affiliated Hospital of Guangzhou Medical University Guangzhou China; ^5^ Department of Thoracic Surgery Shanghai Chest Hospital Shanghai Jiao Tong University School of Medicine Shanghai China; ^6^ Department of Thoracic Surgery The First Affiliated Hospital of Henan Medical University Xinxiang China; ^7^ Dongguan Key Laboratory of Precision Medicine Precision Medicine Center The First Dongguan Affiliated Hospital Guangdong Medical University Guangdong China; ^8^ Department of Gastrointestinal Surgery The Fifth Affiliated Hospital of Guangzhou Medical University Guangzhou China

**Keywords:** esophageal squamous cell carcinoma, immunotherapy combination, metastasis, plasma biomarker, PTM crosstalk

## Abstract

Posttranslational modification (PTM) is pivotal in cancer progression. However, the mechanisms, biological function, and clinical significance underlying PTM crosstalk are unclear. Here, we performed systematic analyses of 2‐hydroxyisobutyrylation (Khib), phosphoproteomic, proteomic, and transcriptomic profiles from 60 esophageal squamous cell carcinoma (ESCC) samples, comprising matched normal, primary tumor, and metastatic lymph node tissues. The integrative analysis identified 4499 proteins co‐modified by Khib and phosphorylation on distinct residues. Interestingly, while the two PTMs target distinct motifs across proteins, they engage common motifs within individual proteins. Functionally, Khib and phosphorylation show prevalent positive crosstalk linked to metastatic progression. Mechanistically, Khib mediates this intra‐protein crosstalk by recruiting kinases and potentiating phosphorylation events. Machine learning‐based artificial intelligence (AI) analysis of secreted proteins with PTM crosstalk revealed an independent and highly effective plasma signature predicting lymph node metastasis. Moreover, molecular subtyping stratified ESCC into three groups, with immunotherapy‐resistant Subtype 3 associated with the worst survival. While combined treatment of integrin inhibitor cilengitide and immunotherapy exhibited targeted efficacy. By deciphering the mechanistic basis of Khib‐phosphorylation crosstalk, identifying a novel plasma biomarker for lymph node metastasis, and providing a therapeutic strategy to sensitize tumors to immunotherapy, this work collectively advances the precision diagnosis and treatment of ESCC.

## Graphical abstract

This study deciphers the functional crosstalk between 2‐hydroxyisobutyrylation (Khib) and phosphorylation in driving esophageal squamous cell carcinoma (ESCC) metastasis. Through multi‐omics and machine learning, we developed a plasma protein signature for noninvasive detection of lymph node metastasis. Furthermore, we defined molecular subtypes and identified a combined therapeutic strategy to overcome immunotherapy resistance.

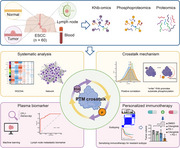

## 1 Introduction

Posttranslational modifications (PTMs) expand proteome functional diversity by adding specific chemical moieties to amino acid side chains [1]. PTMs dynamically influence protein localization, structure, and activity, ultimately modulating diverse aspects of cell biology. Multiple PTMs are commonly integrated at various sites on a single protein, collectively determining its functional outcomes [2]. Emerging evidence indicates that crosstalk between PTMs critically regulates diverse cellular processes, including tumorigenesis [3, 4, 5, 6, 7, 8]. Nevertheless, the specific crosstalk events that drive cancer progression, along with the mechanistic principles governing these complex networks, remain largely unexplored. This constitutes a fundamental gap in our understanding, particularly for prevalent modifications like phosphorylation.

2‐Hydroxyisobutyrylation (Khib) is a form of acylation modification attracting increasing interest for its potential roles in disease [9]. While preliminary evidence has linked Khib to cancer, a comprehensive understanding of its mechanistic contributions and therapeutic potential remains under investigation [10]. Although our previous study identified the substrate profile of Khib, the substrate characteristics and regulatory mechanisms remain to be elucidated [11]. Critically, whether Khib undergoes crosstalk with other PTMs, the biological functions of crosstalk‐associated proteins, and their clinical significance require elucidation. More recently, we performed phosphoproteomic sequencing on the same batch of samples, generating a comprehensive phosphorylation‐substrate map [12]. Integrative analysis of the two datasets uncovers an extensive set of proteins harboring both Khib and phosphorylation. However, it remains unclear whether Khib crosstalks with phosphorylation to regulate metastasis. The inherent complexity of PTM crosstalk holds substantial yet unexplored potential for the discovery of biomarkers and therapeutic targets in cancer [13, 14]. Thus, elucidating the clinical significance of Khib‐phosphorylation crosstalk is essential for translating basic findings into personalized cancer therapy.

Lymph node (LN) metastasis in esophageal squamous cell carcinoma (ESCC) constitutes a significant threat to patient health [15]. Accurate prediction of LN metastasis status is critical for selecting the appropriate surgical approach and prolonging survival [16]. Current methods for evaluating LN metastasis in ESCC patients preoperatively are either invasive or insufficiently accurate [17]. Existing serum biomarkers for predicting LN metastasis in ESCC are suboptimal, and there is a pressing need to develop biomarkers capable of accurate prediction [18]. Artificial intelligence (AI) has become a transformative tool in the development of tumor markers [19]. However, serum biomarkers based on PTM crosstalk proteins and developed using AI for the accurate prediction of LN metastasis in ESCC remain lacking.

Molecular subtyping of ESCC is one of the key strategies for precision medicine aimed at improving patient outcomes [20, 21]. Several studies have identified distinct molecular subtypes of ESCC based on proteomics and further investigated subtype‐specific treatment strategies [22, 23, 24]. However, ESCC still lacks clinically relevant molecular subtypes that can facilitate patient stratification and enhance the efficacy of immunotherapy. Although immune checkpoint inhibitors constitute a significant and effective treatment for ESCC, only a subset of patients derive benefit from them [25]. Combination immunotherapy strategies represent a promising approach to overcoming resistance to immunotherapy [26]. Therefore, there is an urgent need to define new molecular subtypes for ESCC and to investigate effective immunotherapy combinations to improve patient outcomes.

In this study, we aimed to characterize the substrate motifs for Khib and phosphorylation across proteins through multi‐omic profiling and validate the crosstalk between the two PTMs within individual proteins. We also sought to leverage machine learning‐based AI to identify metastatic serum biomarkers and define distinct ESCC molecular subtypes. The personalized treatment strategies will provide critical insights into the management of this life‐threatening disease.

## 2 Results

### 2.1 Khib‐Modified Proteins Orchestrate the Malignant Progression of ESCC

**FIGURE 1:**
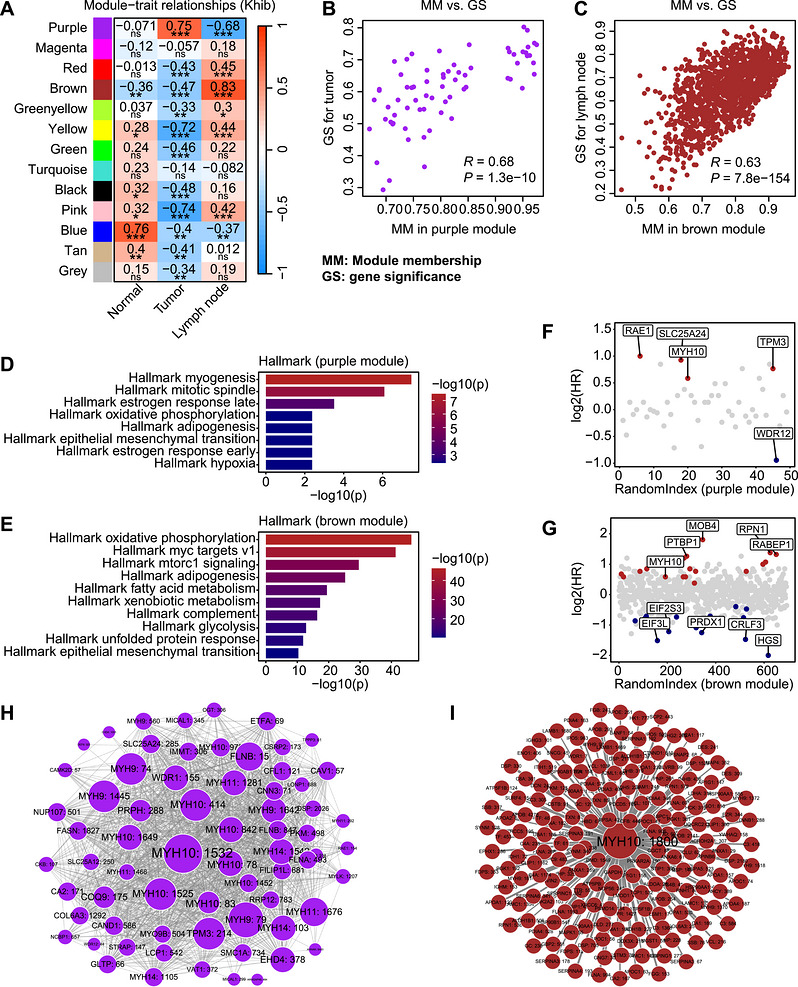
Identification of key Khib‐modified proteins modules in the malignant progression of ESCC. (A) Correlation analysis between module eigengenes and clinical traits. *p*‐value levels are denoted as ns, *p* > 0.05; ^*^, *p* < 0.05; ^**^, *p* < 0.01; and ^***^, *p* < 0.001. (B) The correlation between gene significance (GS) and module membership (MM) in the purple module (tumor‐specific purple module). (C) The correlation between GS and MM in the brown module (LN‐specific brown module). (D) Hallmark gene sets enrichment analysis of the tumor‐specific purple module. (E) Hallmark gene sets enrichment analysis of the LN‐specific brown module. (F) Overall survival analysis of tumor‐specific purple module proteins in protein level. Red: risk factors (hazard ratio > 1, *p* < 0.05), blue: protective factors (hazard ratio < 1, *p* < 0.05). (G) Overall survival analysis of LN‐specific brown module proteins in protein level. Red: risk factors (hazard ratio > 1, *p* < 0.05), blue: protective factors (hazard ratio < 1, *p* < 0.05). (H) Protein–protein interaction network of the tumor‐specific purple module. The interactions were created by the correlation of Khib. The size of the node indicates the connectivity between the proteins. (I) Protein–protein interaction sub‐network of LN‐specific brown module with hub protein MYH10 (P35580). The interactions were created by the correlation of Khib.

To investigate the role of Khib in ESCC progression, the network‐based AI method Weighted Gene Co‐Expression Network Analysis (WGCNA) was applied to Khib proteomic data to identify driver modules in primary tumors and metastatic LNs. By applying the optimal soft threshold, a co‐expression network was established, resulting in 13 modules, visually represented by unique colors (Figure S1A,B). Module‐clinical trait correlation analysis not only showed significant positive correlations between the purple module and tumorigenesis, but also indicated the importance of the brown, red, and green–yellow modules in metastasis (Figure 1A and Figure S1C). Further gene significance‐module membership analysis enabled us to narrow our focus to the purple and brown modules (Figure 1B,C). Of note, these two modules were mainly associated with oxidative phosphorylation and posttranslational protein phosphorylation (Figure 1D,E and Figure S1D–G, and Table S1). Survival analysis based on protein expression profiles showed multiple prognosis‐related proteins in both modules (Figure 1F,G and Figure S1H,I). Interestingly, MYH10 (myosin heavy chain 10) appeared in both modules; in particular, the co‐expression network of the tumor‐specific purple module showed that MYH10 was the hub protein, while the sub‐network of LN‐specific brown module showed that MYH10 had more correlation (Figure 1H,I). These data collectively suggest the critical clinical significance and functional importance of key Khib‐modified proteins in ESCC.

### 2.2 Positive Functional Crosstalk Between Khib and Phosphorylation at Distinct Sites Is Important for ESCC Development

**FIGURE 2:**
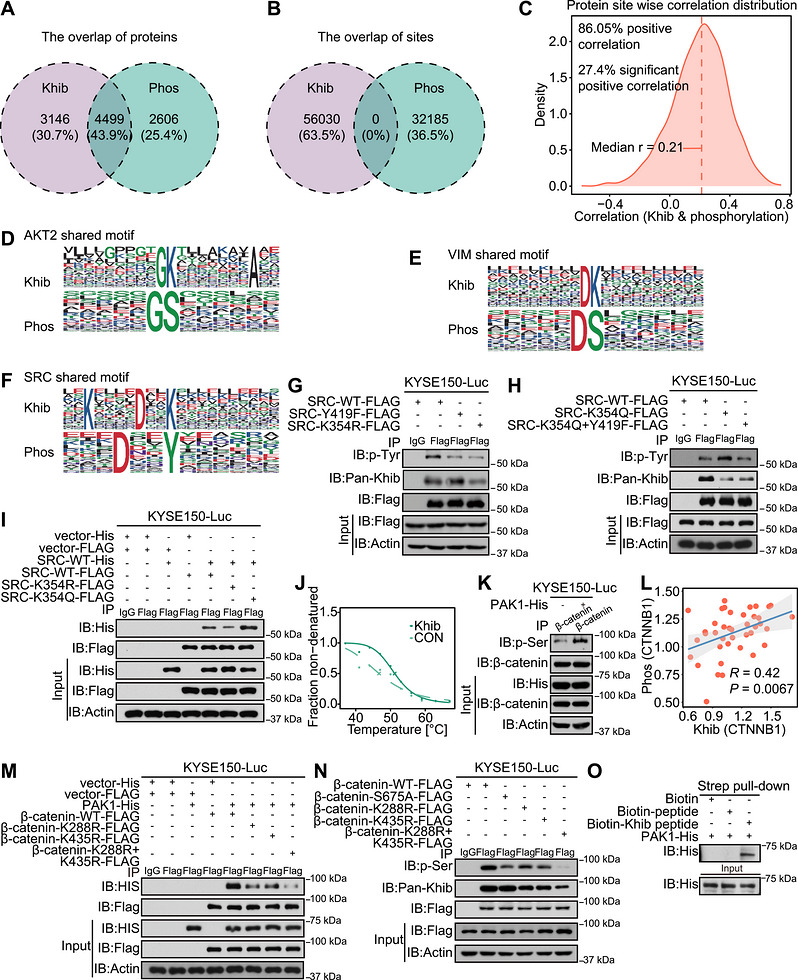
Crosstalk between Khib and phosphorylation is important for ESCC development. (A) The overlapping modification proteins between Khib and phosphorylation. Purple: Khib, blue: phosphorylation. (B) The overlapping modification sites between Khib and phosphorylation. Purple: Khib, blue: phosphorylation. (C) Protein site‐wise correlation of Khib and phosphorylation in all samples. (D) The Khib and phosphorylation motifs of AKT2 belonging to the shared motif. (E) The Khib and phosphorylation motifs of VIM belonging to the shared motif. (F) The Khib and phosphorylation motifs of SRC belonging to the shared motif. (G) Immunoprecipitation results revealed differences in p‐Tyr and Khib levels among SRC‐WT, SRC‐K354Q, and SRC‐K354Q‐Y419F mutants. (H) Immunoprecipitation results showed that there were differences in the levels of p‐Tyr and Khib among different SRC mutants. (I) Effects of mutants at distinct Khib modification sites on SRC self‐binding. (J) Shifted thermal stability curves of PAK1 quantified from the MS‐based profiling experiment. (K) PAK1 significantly enhanced the phosphorylation level of β‐catenin. (L) Significantly positive correlation between β‐catenin phosphorylation and Khib levels. (M) Effect of Khib modifications at β‐catenin K288 and K435 sites on PAK1 binding affinity. (N) Immunoprecipitation results indicated differences in p‐Ser and Khib levels among β‐catenin mutants. (O) Immunoblot analysis was performed for the in vitro streptavidin pull‐down assay using a biotin‐labeled Khib peptide and PAK1.

To determine the crosstalk between Khib and phosphorylation, phosphoproteomics data were generated from the same cohort used for Khib proteomic profiling. When analyzed irrespective of tissue type of these 60 ESCC samples, 81.4% have multiple Khib sites and 73.2% contain more than one phosphorylation site (Figure S2A,B). Integrative analysis showed 4499 proteins were co‐modified by Khib and phosphorylation (Figure 2A). Although Khib and phosphorylation sites did not overlap, a global positive correlation between these two PTMs was observed across all tissues (Figure 2B,C).

The 10 most conserved Khib modification motifs flanking lysine (K) sites were identified in ESCC (Figure S2C). Intersection analysis identified 10 conserved motif pairs shared between Khib and phosphorylation, suggesting a potential mechanism for their crosstalk (Table S2). To explore potential crosstalk between Khib and phosphorylation, AKT2 (AKT serine/threonine kinase 2) [27], VIM (vimentin) [28], and SRC (SRC proto‐oncogene, non‐receptor tyrosine kinase) [29] were selected for study owing to their established involvement in diverse oncogenic processes. All these three proteins are co‐modified with Khib and phosphorylation, with each harboring unique paired motifs for these two PTMs (Figure 2D–F). The reciprocal regulation between Khib and phosphorylation was evident in AKT2 and VIM. Specifically, the AKT2‐K165R and VIM‐K168R Khib‐site mutants (lysine to arginine, mimicking the de‐Khib state) showed reduced phosphorylation, whereas the AKT2‐S34A and VIM‐S430A (serine to alanine, phosphor‐inactive mutant) phosphorylation‐site mutants exhibited decreased Khib levels (Figure S2D,E). Furthermore, the Boyden chamber invasion assay showed that AKT2 or VIM overexpression enhanced ESCC cell invasion, while the corresponding modifier‐inactivating mutations (AKT2‐S34A/AKT2‐K265R, VIM‐S430A/VIM‐K268R) do not exhibit this phenomenon (Figure S2F,G). For SRC, the K354R (lysine to arginine, mimicking the de‐Khib state) mutation reduced phosphorylation, whereas Y419F (tyrosine to phenylalanine, phosphor‐inactive mutant) mutation had no effect on Khib modification (Figure 2G). An increase in SRC phosphorylation was observed in the SRC‐K354Q (lysine to glutamine, constitutive active) mutant. This effect was abolished by the SRC‐Y419F mutation (Figure 2H). Mechanistically, SRC undergoes autophosphorylation [30], and the self‐binding of SRC was confirmed by co‐immunoprecipitation (Co‐IP). Notably, the K354R mutant weakened this interaction, while the K354Q mutant significantly enhanced it (Figure 2I), demonstrating that Khib modification at K354 significantly promotes autophosphorylation by facilitating SRC self‐interactions. More importantly, overexpression of SRC‐WT led to a significant increase in cell invasion phenotypes, while a reduction was detected in SRC modifier‐inactivating mutations (Y419F, K354R, and K354Q+Y429F) (Figure S2H).

To further investigate the mechanisms underlying Khib‐phosphorylation crosstalk, thermal proteome profiling was employed to identify proteins capable of recognizing Khib modification. Interestingly, multiple kinases were predicted to interact with Khib modifications, with PAK1 (P21 [RAC1] activated kinase 1) showing particularly high affinity for the modification (Figure 2J). β‐Catenin was identified as a substrate of PAK1 (Figure 2K) and carries a number of Khib modifications, which exhibit significant positive correlation with phosphorylation (Figure 2L). Khib‐site mutations at K288 and K435 markedly diminished PAK1 binding to β‐catenin (Figure 2M), which may account for the significantly lower phosphorylation (Figure 2N). In line with this, in vitro pull‐down assays demonstrated that PAK1 directly binds to the β‐catenin Khib modification (K288) peptide (Figure 2O). To explore the relationship between the crosstalk of these modification sites and their functions, a Boyden chamber invasion assay was conducted. The results showed that overexpression of β‐catenin‐WT significantly promoted ESCC cell invasion, but the modified‐inactive mutant form of β‐catenin (S675A, K288R, and K435R) did not have this effect (Figure S2I). Collectively, these results demonstrate that positive crosstalk between Khib and phosphorylation is a common phenomenon, as evidenced by its presence on a series of key tumor‐associated proteins including AKT2, VIM, and SRC.

### 2.3 The Khib‐Phosphorylation Crosstalk of MYH10 and SMC1A Is Essential for ESCC Progression

**FIGURE 3:**
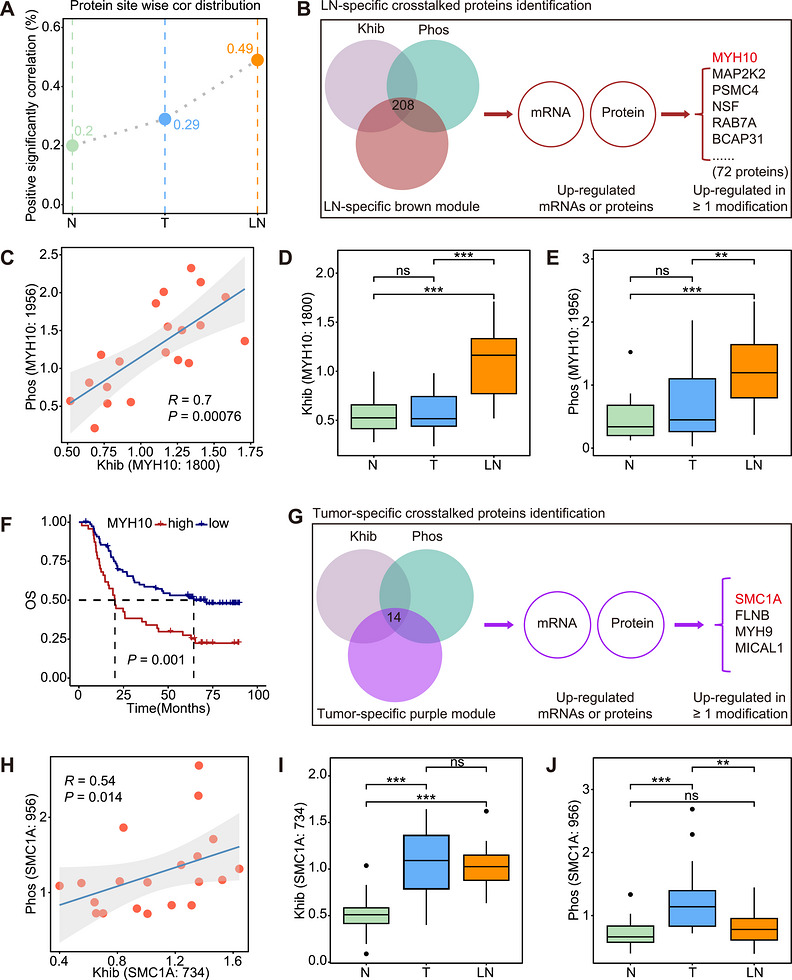
Identification of the key genes with co‐modification. (A) The percentage of significant positive correlations in N, T, and LN groups. (B) Identification of key crosstalk proteins between Khib and phosphorylation in LN‐specific brown module. (C) The significant positive correlation between Khib and phosphorylation of MYH10. (D and E) The Khib (D) and phosphorylation (E) level of MYH10 were upregulated in LN. (F) Kaplan–Meier curves of OS (log‐rank test: *p*  =  0.001) according to MYH10 (P35580) protein expression. (G) Identification of key crosstalk proteins between Khib and phosphorylation in tumor‐specific brown module. (H) The significant positive correlation between Khib and phosphorylation of SMC1A. (I and J) The Khib (I) and phosphorylation (J) level of SMC1A were upregulated in tumor. *P* value levels are denoted as ns, *P* > 0.05; *P < 0.05; **P < 0.01; and ***P < 0.001.

To investigate the Khib‐phosphorylation crosstalk during ESCC metastasis, phosphorylation of proteins from the LN‐specific brown module was assessed across normal (N), tumor (T), and metastatic LN samples. The analysis revealed increased phosphorylation of proteins in LNs, compared with normal tissues (Figure S3A). Analysis of 4499 co‐modified proteins revealed that Khib and phosphorylation exhibited the strongest positive correlation in LN tissues, compared to normal and primary tumor tissues (Figure 3A). From 695 Khib‐modified proteins in the LN‐specific brown module, 72 were identified that were co‐modified by phosphorylation, with a subset upregulated at the mRNA or protein level, and exhibiting increased Khib and/or phosphorylation in LNs (Figure 3B and Table S3). Through functional enrichment analysis, the 72 proteins were found to be associated with core processes including protein folding, translation, and intracellular protein transport (Figure S3B and Table S4). Interestingly, a strong positive correlation was observed between Khib at K1800 and phosphorylation at S1956 on MYH10 (Figure 3C). Moreover, high MYH10 expression was associated with poor survival in ESCC patients. In particular, MYH10 was uniquely identified by its LN‐specific upregulation at the Khib and phosphorylation levels, independent of transcriptional changes (Figure 3D–F and Figure S3C,D). The Boyden chamber invasion assay showed that MYH10‐WT overexpression enhanced ESCC cell invasion, while MYH10‐S1956A and MYH10‐K1800R significantly abrogated these effects (Figure S3E).

Given the prevalence of phosphorylation in the tumor‐specific purple module (Figure S3F), the crosstalk between Khib and phosphorylation in ESCC tumorigenesis was investigated. With the same selection criteria applied to the LN‐specific brown module, four tumor‐specific purple module proteins were found to show elevated mRNA and/or protein expression, along with increased Khib and/or phosphorylation (Figure 3G and Table S3). Among these proteins, SMC1A (structural maintenance of chromosomes 1A) exhibited an exclusively significant positive correlation between Khib modification (K734) and phosphorylation (S956) (Figure 3H). Interestingly, SMC1A exhibits higher levels of Khib modification (K734), phosphorylation (S956), mRNA, and protein levels in tumors (Figure 3I,J and Figure S3G,H). Collectively, these findings suggest Khib‐phosphorylation crosstalk is essential for ESCC progression.

### 2.4 CFL1 and PAFAH1B2 Serves as Noninvasive Biomarkers for LN Metastasis in ESCC

**FIGURE 4:**
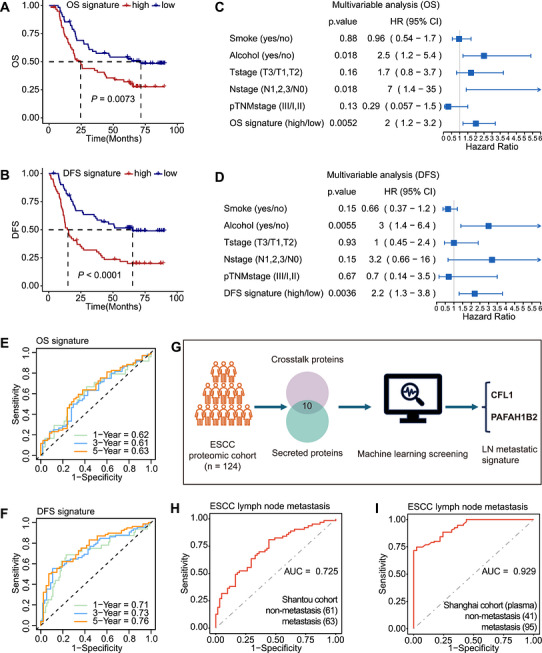
Crosstalk‐based protein prognostic signatures were identified in ESCC. (A and B) Kaplan–Meier curves of OS (log‐rank test: *p*  =  0.0073) (A) and DFS (log‐rank test: *p*  =  0.0001) (B) according to the crosstalk‐based protein signatures. (C and D) Multivariable Cox regression analysis of OS (C) and DFS (D) signatures. (E and F) Time‐dependent ROC analysis for predicting OS (E) and DFS (F) at 1, 3, and 5 years. (G) Workflow of identifying LN metastatic signature. (H and I) ROC analysis for predicting LN in training set (H) and validation set (I).

To investigate the prognostic significance of Khib‐phosphorylation co‐modified proteins, univariate Cox analysis of a public proteomic cohort (Shantou cohort) [23] identified proteins associated with overall survival (OS) (MYH10 and ARHGDIA) and disease‐free survival (DFS) (TPD52L2, HSPD1, HNRNPK, and HNRNPU) (Figure 3F and Figure S4A–E). Based on these proteins, patients were stratified into high‐ and low‐risk groups based on signature scores derived from Cox regression coefficients, respectively, and the high‐risk group exhibited significantly reduced OS and DFS (Figure 4A,B). Univariate and multivariate analyses confirmed that both signatures were independent risk factors, even after adjusting for clinical variables (Figure 4C,D and Figure S4F,G). Model performance was assessed by ROC analysis, with 1‐, 3‐, and 5‐year AUCs of 0.62, 0.61, and 0.63 obtained for the OS signature, and 0.71, 0.73, and 0.76 for the DFS signature (Figure 4E,F), demonstrating the strong predictive potential of these signatures.

To identify biomarkers for LN metastasis, the secreted proteins with co‐modification were prioritized for further analysis (Figure 4G). From the 12 differentially expressed secreted proteins identified from the 72 proteins in the LN‐specific brown module, 10 valuable proteins were identified by least absolute shrinkage and selection operator (LASSO) logistic regression analysis (Figure S4H,I). CFL1 (cofilin 1) and PAFAH1B2 (platelet‐activating factor acetylhydrolase 1b catalytic subunit 2) were identified as independent predictors of LN metastasis by subsequent univariate and multivariate logistic regression analyses of these 10 proteins (Figure S4J,K). A logistic regression model was constructed using CFL1 and PAFAH1B2 protein expression to distinguish ESCC patients with LN metastasis from nonmetastatic individuals. This two‐proteins‐based predictive signature showed great performance in distinguishing ESCC LN from nonmetastatic samples with an AUC of 0.725 in Shantou cohort (Figure 4H). To evaluate the translational potential of the predictive signature, the expression of these proteins was assessed by enzyme‐linked immunosorbent assay (ELISA) in a clinical serum cohort comprising 136 ESCC patients (Table S5). The predictive signature constructed using the same coefficients for these two proteins, was evaluated in the validation set and demonstrated high predictive accuracy, with an AUC of 0.929 (Figure 4I). Of note, as a serum protein‐based biomarker, our signature outperformed the current serum LN metastasis protein markers squamous cell carcinoma antigen (SCC‐Ag) (Figure S4L) and CA125 (AUC = 0.57) [31], providing potential noninvasive biomarkers for LN metastasis in ESCC.

### 2.5 Molecular Subtyping Identifies Three ESCC Subtypes Defined by Crosstalk Proteins With Divergent Prognosis and Distinct Therapeutic Vulnerabilities

**FIGURE 5:**
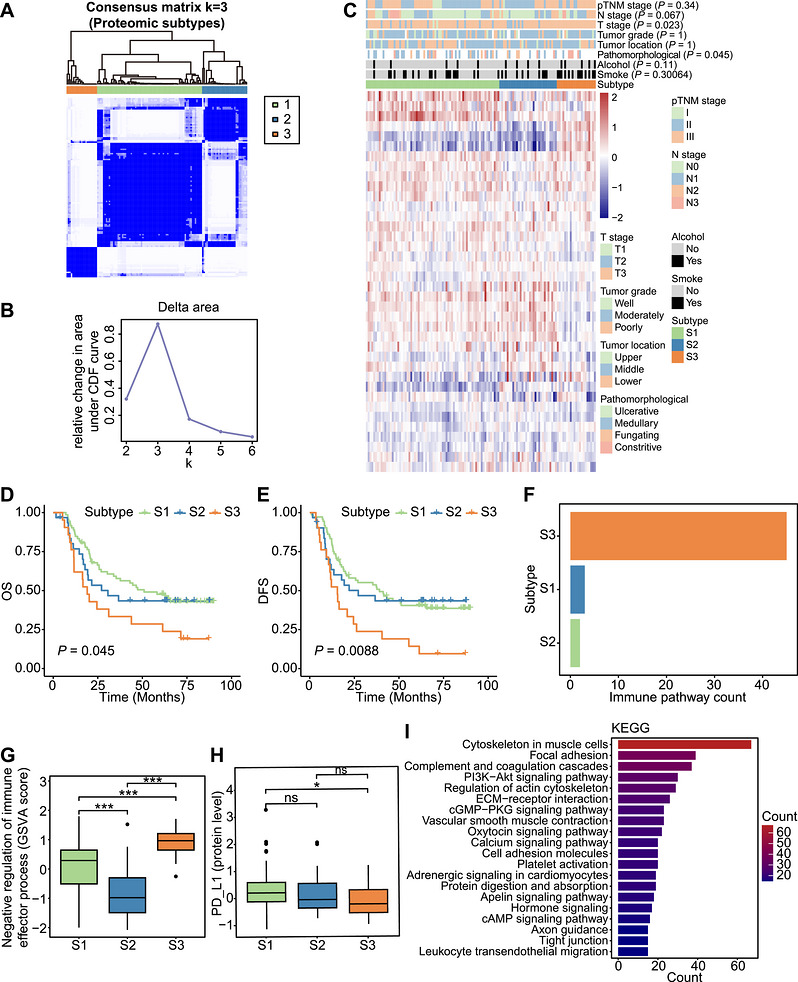
Three molecular subtypes of ESCC were defined based on crosstalk proteins. (A) The consensus matrix of all samples with three proteomic clusters (*K* = 3). The heatmap shows the similarity of ESCC patients. (B) The delta area indicated that the optimal cluster number was obtained when *K*  =  3. (C) Heatmap depicting the protein abundance of crosstalk proteins. Pathomorph: Pathomorphological. (D and E) Kaplan–Meier curves of OS (log‐rank test: *p* = 0.045) (D) and DFS (log‐rank test: *p*  =  0.0088) (E) for three subtypes. (F) The counts of upregulated immune pathways for each subtype. (G) Negative regulation of immune effector process pathway was upregulated in Subtype 3. (H) The protein level of PD‐L1 was downregulated in Subtype 3. (I) KEGG enrichment of upregulated proteins belonging to Subtype 3. *P* value levels are denoted as ns, *P* > 0.05; *P < 0.05; **P < 0.01; and ***P < 0.001.

Proteomics‐based molecular subtypes have been extensively used to guide patient stratification and personalized treatment in other cancer types [32, 33, 34]. We next sought to investigate whether the ESCC samples could be stratified into clinically relevant molecular subtypes based on Khib‐phosphorylation co‐modified proteins, including 4 from tumor‐specific purple module and 72 from LN‐specific brown module proteins (Figure 3B,G). Consensus clustering of the Shantou cohort generated three ESCC subtypes (S1: *n* = 72; S2: *n* = 31; and S3: *n* = 21) (Figure 5A,B and Figure S5A). Clinical annotation revealed that advanced T stage (*p* = 0.023) was more prevalent in S3 than in other two subtypes (Figure 5C). Accordingly, patients classified as S3 had worse OS and DFS outcomes compared with S1 and S2 (Figure 5D,E). To further characterize these three subtypes, differential expression analysis of all proteins was conducted across the 124 tumor samples from the Shantou cohort. S1 was identified as a collagen‐related subtype, based on the enrichment of its upregulated proteins in pathways for collagen‐containing extracellular matrix formation and metabolism (Figure S5B and Table S6). Upregulated proteins in S2 were enriched for pathways involved in chromosome segregation, nuclear division, and cell cycle, thus characterizing it as a cell cycle‐related subtype (Figure S5C and Table S6). Subtype S3 was selected for further analysis due to its association with the worst survival.

Compared to S1 and S2, proteins in Subtype S3 showed significantly more enrichment in immune‐related pathways (Figure 5F). Furthermore, negative regulation of immune effector process signaling was increased, suggesting an immunosuppressive phenotype in the S3 subgroup (Figure 5G). Paradoxically, PD‐L1 protein levels were reduced in S3 compared with S1, indicating that S3, while immune‐enriched, might have a diminished response to immunotherapy (Figure 5H). To further validate immune characteristics, the pathway activity for cancer hallmark‐related pathways in all patients was calculated based on gene set variation analysis (GSVA) (Figure S5D). In line with the results of functional enrichment analysis, immune‐related hallmarks were more active in S3 (Figure S5D). KEGG enrichment analysis was performed to explore potential therapeutic strategies. The focal adhesion signaling pathway was found to be enriched in the S3 subtype (Figure 5I and Table S6), indicating that targeting this signaling pathway, either alone or in combination with immunotherapy, may be beneficial for S3 patients. Collectively, these results suggest that the crosstalk proteins are helpful in identifying the molecular subtypes of ESCC and exploring potential treatment strategies.

### 2.6 Focal Adhesion Pathway Inhibitor Enhances Immunotherapy Efficacy in the Immunotherapy‐Resistant ESCC Subtype

**FIGURE 6:**
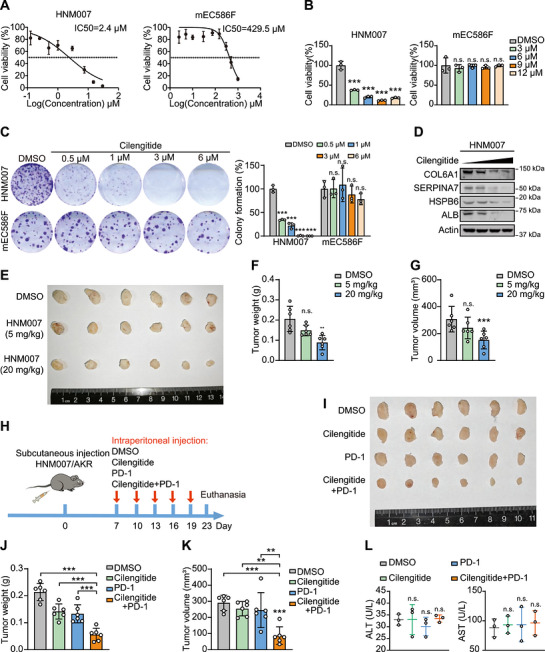
Cilengitide enhances immunotherapy efficacy in vitro and in vivo. (A) The 50% inhibitory concentration (IC50) of cilengitide was determined in HNM007 and mEC586F cell lines. (B and C) Cell viability assay (B) and colony formation assay (C) were performed to evaluate the effect of cilengitide on the proliferation of HNM007 and mEC586F cells. (D) The effect of cilengitide on the expression of S3 subtype signature proteins was validated by Western blotting. (E) Representative image of tumors from the control group and cilengitide‐treated groups. (F and G) Bar graphs showing tumor volume (F) and weight (G) demonstrate that cilengitide significantly inhibits tumor growth. (H) Mice‐bearing HNM007/AKR xenografts tumor were administered cilengitide and/or anti‐PD‐1 every 3 days. (I) Representative image of tumors from the control and drug‐treated group. (J and K) Bar graphs showing tumor volume (J) and weight (K) indicate that the combination of cilengitide and anti‐PD‐1 significantly suppressed tumor growth. (L) Key toxicity indicators were detected in mice treated with indicated inhibitors or DMSO. *P* value levels are denoted as ns, *P* > 0.05; *P < 0.05; **P < 0.01; and ***P < 0.001.

To investigate potential therapeutic strategies for Subtype S3, we used HNM007 and AKR cell lines as models of the S3 subtype due to their established resistance to immunotherapy in murine esophageal cancer, with mEC586F cells serving as a sensitive control [35]. In this study, we selected cilengitide, which specially targets integrins, the key initiators of the focal adhesion signaling pathways. The cell viability assay showed that HNM007 and AKR cells were more sensitive to cilengitide than mEC586F cells (Figure 6A and Figure S6A). In line with this, the viability of HNM007 and AKR cells was markedly suppressed by cilengitide in a dose‐dependent manner, while mEC586F cells exhibited minimal response (Figure 6B and Figure S6B). Moreover, cilengitide exclusively inhibited colony formation in HNM007 and AKR cells in a dose‐dependent manner, leaving mEC586F cells unaffected (Figure 6C and Figure S6C). As expected, cilengitide dose‐dependently suppressed the expression of representative genes in S3 subtype, including COL6A1, SERPINA7, HSPB6, and ALB (Figure 6D and Figure S6D). Furthermore, the in vivo growth of HNM007 tumors was significantly suppressed by intraperitoneal administration of cilengitide at a dose of 20 mg/kg (Figure 6E–G). These results demonstrate that cilengitide can effectively target the S3 subtype to treat esophageal cancer.

To assess the potential of cilengitide to overcome immunotherapy resistance in the S3 subtype, HNM007 and AKR tumor‐bearing mice were treated with cilengitide and/or anti‐PD‐1 every 3 days (Figure 6H). Notably, while monotherapies showed slight anti‐tumor activity, the combination therapy resulted in significant reduction in tumor growth (Figure 6I–K and Figure S6E–G). To assess the systemic toxicity of cilengitide and/or anti‐PD‐1, morphological changes in major organs and hematological parameters were examined. No significant alterations were observed between treated and untreated groups (Figure 6L and Figure S6H). In conclusion, these results indicate that cilengitide enhances antitumor immunity induced by PD‐1 blockade, providing a promising therapeutic strategy for the immunotherapy‐resistant patient population.

## 3 Discussion

Although tumor metastasis is the leading cause of cancer‐related mortality, its molecular mechanisms remain incompletely understood [36, 37, 38, 39, 40, 41, 42, 43, 44]. Crucially, existing studies suggest that gene amplification and mutations alone are insufficient to explain tumor metastasis, underscoring the pivotal role of PTMs in this process [11, 12, 45, 46]. In the context of ESCC management, LN status critically guides ESCC treatment: endoscopic resection suffices for node‐negative disease, whereas radical lymphadenectomy improves survival by clearing metastatic foci in node‐positive patients [17]. Notably, overly extensive lymphadenectomy increases postoperative morbidity, adversely impacting quality of life. Current methods like CT imaging and LN biopsy have limitations in accuracy or invasiveness, highlighting an unmet need for noninvasive biomarkers [18]. Moreover, previous single‐gene or protein biomarkers are inadequate for capturing the regulatory complexity introduced by PTM crosstalk, thereby limiting their clinical utility [47, 48]. To address these gaps, we leveraged AI to develop a multi‐protein‐based blood biomarker for noninvasive prediction of LN metastasis in ESCC, which incorporates PTM crosstalk information. More importantly, multi‐protein combination biomarkers demonstrate greater stability than single‐protein biomarkers [49, 50, 51, 52]. Collectively, our established noninvasive biomarkers represent clinically applicable tools for LN metastasis prediction and potential therapeutic targets for precision oncology.

While the occurrence of PTM crosstalk has been observed in various biological processes, its specific functional roles in driving pathological states like cancer metastasis remain poorly delineated, let alone the molecular mechanisms that govern these interactions [2, 3, 4]. Addressing this gap, we present the first study investigating the Khib‐phosphorylation crosstalk mechanism from the perspectives of substrate characteristics, crosstalk effects, and molecular mechanisms. Mechanistically, widespread crosstalk with positive correlations between Khib and phosphorylation sites was observed on individual proteins. In addition, the conserved motif we identified provides specific evidence of Khib‐phosphorylation crosstalk in ESCC. Recent studies have indicated that crosstalk frequently occurs at adjacent PTM sites [53] and may regulate metabolism‐related pathways [54]. Focusing on proteins harboring shared motifs within oncogenic pathways, we demonstrated that Khib modification facilitates recruitment of specific protein kinases, thereby enhancing substrate phosphorylation. Notably, phosphorylation, as the most common PTM, is closely associated with tumorigenesis through crosstalk with other modifications [5, 55]. However, despite the central role of Khib in tumor progression, crosstalk between Khib and phosphorylation had remained unexplored prior to this study. By comprehensively characterizing this crosstalk in multi‐tissue samples, we elucidate its patterns and regulatory mechanisms. Overall, these findings establish a foundational resource for PTM crosstalk research and elucidate the mechanism of Khib‐phosphorylation crosstalk.

Despite extensive research on molecular subtyping of esophageal cancer, including genomic [20], transcriptomic [21], there is no established classification system in clinical practice to guide treatment, particularly for immunotherapy. Our analysis based on crosstalk proteins categorizes ESCC patients into three distinct molecular subtypes, providing guidance for clinical immunotherapy. Collagen‐associated S1 and cell cycle‐related S2 subtypes were defined by clinical and pathway characterization across molecular subtypes. It has been documented that excessive collagen deposition enhances matrix stiffness, reduces vascular permeability, and stimulates integrin‐dependent signaling to drive tumor progression [56]. Accordingly, patients in S1 subtype may benefit from therapies that decrease TME collagen, such as the vitamin A analog all‐trans retinoic acid, which targets cancer‐associated fibroblasts (CAFs) to suppress collagen production [57]. CDK4/6 inhibitors, such as palbociclib, may offer a promising targeted therapy for S2 subtype patients. S3 patients exhibit poorest survival with immunotherapy resistance, underscoring the necessity for novel combinatorial approaches. Recent studies have highlighted that the combination of integrin inhibitors and immunotherapy possesses multiple promising mechanisms in clinical cancer treatment [58]. Moreover, accumulating evidence suggests that integrin inhibition may sensitize tumors to immunotherapy [59, 60]. Given the enrichment of focal adhesion pathway in S3 subtype, we performed multi‐step screening to identify potential therapeutic strategies. This revealed that the focal adhesion inhibitor cilengitide enhances immunotherapy efficacy in S3 models. Consequently, targeting this subtype provides a strategy to overcome immunotherapy resistance and offers a precision medicine approach through combination therapies. Collectively, our findings provide new insights into the clinical molecular classification of ESCC, with direct implications for developing subtype‐specific immunotherapy combinations.

Several limitations of this study should be acknowledged. Due to the lack of known Khib regulatory enzymes, direct validation of the regulatory mechanisms underlying the bidirectional versus unidirectional effects among AKT2, VIM, and SRC remains challenging. In addition, the plasma validation cohort was derived from a single center, and future studies incorporating multicenter cohorts are needed to further validate the clinical utility of the identified signature. Moreover, the sample size of the serum validation cohort is relatively limited, and larger cohorts are required to confirm the generalizability of the biomarker.

In conclusion, based on systematic analysis of multi‐omics PTM data, we elucidated the key mechanism underlying Khib‐phosphorylation crosstalk. Using crosstalk proteins, we leveraged machine learning‐based AI to develop a robust serum signature for cancer metastasis and stratified ESCC into three molecular subtypes, identifying effective immunotherapy combinations for immunotherapy‐resistant Subtype 3. These findings have important implications for PTM crosstalk research and precision cancer diagnosis and treatment.

## 4 Materials and Methods

This study adhered to the REporting recommendations for tumor MARKer prognostic studies (REMARK) guideline for diagnostic/prognostic studies (Table S7).

### 4.1 Data Collection and Processing

Matched normal tissues, primary tumor tissues, and matched LN tissues from 20 ESCC patients were collected as previously described [11]. In these samples, the raw and processed data for Khib, phosphoproteomic, proteomic, and transcriptomic profiling were obtained from our two previous studies [11, 12]. Briefly, in this study, we leveraged the preprocessing and differential analysis results of all these omics data from our previous studies. The public ESCC proteomics cohort, containing complete OS and DFS information, was utilized to construct our signatures and molecular subtypes [23]. The public ESCC proteomics cohort comprises 124 paired samples, and the proteomic LC–MS/MS data were normalized and log2 transformed. A clinical serum cohort comprising 136 ESCC patients was obtained from Shanghai Chest Hospital, Shanghai Jiao Tong University for ELISA.

All missing values were imputed using the R package impute (v 1.82.0) with the parameter *k* = 5. For public proteomic datasets and our log2‐transformed proteomic data, missing values were imputed using k‐nearest neighbors algorithm. Similarly, missing values in the Khib‐site profile and phosphorylation‐site profile were imputed using the same method and parameters. The protein Khib levels were calculated based on the median ratio of Khib sites within the same proteins. The method for calculating protein phosphorylation levels mirrors that used for Khib.

### 4.2 Weighted Correlation Network Analysis

We used the R package WGCNA (v1.73) to construct a co‐expression network based on the Khib modification profile in ESCC. The Khib‐site profile, with missing values imputed, was used as input data for WGCNA. After assessing the sample clusters, no outliers were detected. Subsequently, the pickSoftThreshold function was employed to determine the appropriate soft threshold and construct the network. A power of 16 was selected as the optimal soft‐thresholding value, as it was the lowest value at which the scale‐free topology fit index curve reached a relatively high value and began to plateau. Module merging was conducted using a mergeCutHeight parameter of 0.25. To identify modules associated with tumors and LN metastasis, we selected modules with significant positive correlations for further analysis. Cytoscape (v 3.10.3) was employed for visualizing protein interactions in the tumor and LN metastasis modules. Spearman's correlation coefficient was used to calculate correlations between different sites within these modules. *p*‐values were adjusted using the Benjamini–Hochberg (BH) method. Sites with adjusted *p*‐values < 0.05 in each module were deemed significantly correlated and utilized for network construction and visualization.

### 4.3 Functional Analysis of Modules

Functional enrichment analysis of modules was conducted using the Metascape online tool to identify enriched GO biological processes, KEGG pathways, hallmark‐related pathways, and Reactome gene sets. A protein list mapped to gene symbols was used as input for Metascape. The analysis was performed using Metascape's default parameters: min overlap = 3, *p*‐value cutoff = 0.01, and min enrichment = 1.5.

### 4.4 Motif Analysis

The MoMo analysis tool, based on the motif‐x algorithm, was used to analyze the motif characteristics of modified sites. Peptide sequences comprising 10 amino acids upstream and downstream of each identified modified site (6 amino acids upstream and downstream for phosphorylation modifications) were used as analysis inputs. The analysis background included peptide sequences comprising 10 amino acids upstream and downstream of all potential modification sites in the species (6 amino acids upstream and downstream for phosphorylation modifications). A sequence pattern was considered a motif of the modification site when the number of peptide segments exhibiting the pattern exceeded 20 and the statistical test *p*‐value was less than 0.000001.

### 4.5 Development of LN Metastasis and Prognostic Signatures via Machine Learning‐Based AI Approaches

To establish serum biomarkers predicting LN metastasis in ESCC patients, we first selected a subset of secreted proteins from LN‐specific brown module crosstalk proteins using annotations from the HPA and Uniprot databases. Intersection with differentially expressed proteins from an internal cohort identified 12 differentially expressed secreted proteins. Subsequently, LASSO analysis (with the *λ*_min_ value determined by 10‐fold cross‐validation) and univariate logistic regression analysis (*p* < 0.05) identified two key secreted proteins. Multivariate logistic regression analysis demonstrated that both were independent factors. Finally, the signature for each patient was calculated as the sum of the products of the multivariate logistic regression coefficients and protein expression levels.

For OS and DFS, the signature development procedure is as follows: (a) univariate Cox regression identifies proteins associated with prognosis in the public ESCC proteomics cohort and (b) multivariate Cox regression establishes the predictive signature. The independence between clinical factors (Smoke [yes/no], Alcohol [yes/no]), T stage (T3/T1 and T2), N stage (N1, N2, and N3/N0), and pTNM stage (III/I and II)) and the signature was assessed using univariate and multivariate Cox proportional hazards models. Significance at *p* < 0.05 was considered indicative of an independent predictive factor. The time‐dependent ROC curve for the signature was generated using the R package timeROC (v 0.4).

### 4.6 Crosstalk Protein‐Based Proteomic Subtyping Analysis

#### 4.6.1 Molecular Subtypes (Consensus Clustering)

The protein expression data from tumor samples in the public ESCC proteomics cohort were employed to identify molecular subtypes via the R package ConsensusClusterPlus (v 1.72.0). Prognostic‐related proteins in the tumor‐specific purple module and LN‐specific brown module were utilized for consensus clustering. The consensus clustering parameters were set as follows: maxK = 6, reps = 1000, clusterAlg = “pam,” and distance = “pearson.” Subsequently, the optimal *K* value for consensus clustering was determined to be 3 using the consensus score matrix and CDF curve. Thus, the ESCC patients were finally clustered into three molecular subtypes (S1, S2, and S3).

#### 4.6.2 Clinical Factors Comparison of Subtypes

The Chi‐square test or Fisher's exact test was employed to assess the differences in clinical characteristics between S3 and other subtypes for 2 × 2 contingency tables.

#### 4.6.3 Differential Protein Analysis Across Three Subtypes

To identify proteins upregulated in each subtype, we conducted differential expression analysis. The R package limma (v 3.64.1) was employed to compare protein expression differences between each subtype and all other subtypes. Proteins with BH‐corrected *p*‐values < 0.01 and fold changes > 1.5 were designated as significantly upregulated in the subtype, that is, subtype‐specific proteins.

#### 4.6.4 Subtype Signature Proteins

To identify proteins predictive of the S3 subgroup, proteins upregulated in the S3 subgroup were selected for further analysis. The random forest machine learning method, implemented via the R package randomForest (v 4.7‐1.2), was employed to assess the importance of these proteins in differentiating the S3 subgroup from other subgroups. The function “importance” was employed to calculate variable importance scores. The top 10 proteins ranked by these scores were designated as signature proteins for the S3 subgroup. Subsequently, predictive performance analysis was conducted for each protein, with ROC analysis revealing that the AUC for all proteins exceeded 0.9.

#### 4.6.5 Functional Enrichment Analysis

Subtype functional enrichment analysis was conducted using the R package clusterProfiler (v 4.16.0). Proteins significantly upregulated in subtypes were used as input for hypergeometric distribution analysis of GO, KEGG, and REACTOME pathways.

#### 4.6.6 Gene Set Variation Analysis

To quantify pathway activity for each patient subgroup, we employed the R package GSVA (v 2.2.0). Cancer hallmark‐related pathways were retrieved from the MSigDB database, and immune‐related pathways were retrieved from the ImmPort database.

### 4.7 Cell Culture and Drug Treatment

The human ESCC cell line KYSE150 was obtained from DSMZ (Braunschweig, Germany) and cultured in RPMI 1640 medium (Thermo Fisher Scientific, Waltham, MA, USA) supplemented with 10% fetal bovine serum (FBS; ExCell Bio, Shanghai, China) at 37°C with 5% CO_2_. The mouse HNM007, AKR, and mEC586 cells were kindly provided by Dr. Anil Rustgi from Columbia University and cultured in Dulbecco's modified Eagle's medium (DMEM) medium (Thermo Fisher Scientific, Waltham, MA, USA) supplemented with 10% FBS at 37°C with 5% CO_2_. All cell lines used were cultured within 35 generations and tested negative for *Mycoplasma* throughout the study.

### 4.8 Western Blot

Cell lysates were mixed with loading buffer, heated, and separated by sodium dodecyl sulfate‐polyacrylamide gel electrophoresis (SDS‐PAGE). Proteins were transferred to a polyvinylidene fluoride (PVDF) membrane, which was then blocked with 5% nonfat milk in Tris‐buffered saline containing Tween‐20 (TBST). The membrane was incubated with primary antibodies for 1–2 h at room temperature, washed with TBST, and subsequently probed with a horseradish peroxidase (HRP)‐conjugated secondary antibody for 1 h at room temperature. Signals were detected using the Clarity Western ECL Substrate Kit (Bio‐Rad, Hercules, CA, USA). Primary antibodies included pan‐specific antibodies targeting lysine Khib modifications, pan‐specific antibodies targeting tyrosine phosphorylation modifications (PTM Biolab, Hangzhou, China), as well as antibodies against Flag (Sigma‐Aldrich, St. Louis, MO, USA), pan‐specific antibodies targeting serine phosphorylation modifications (Santa Cruz Biotechnology, Santa Cruz, CA, USA), β‐actin, His, COL6A1, SERPINA7, HSPB6, and ALB (Proteintech, Chicago, IL, USA).

### 4.9 Co‐Immunoprecipitation

For Co‐IP assays, cell lysates were precleared with IgG (Santa Cruz Biotechnology) and protein A/G Sepharose beads (Santa Cruz Biotechnology) for 1 h at 4°C. The supernatant was incubated with a specific primary antibody overnight at 4°C, followed by incubation with protein A/G beads for 4 h. Beads were washed with phosphate‐buffered saline (PBS) and lysis buffer, then resuspended in 5× SDS loading buffer for subsequent Western blot analysis.

### 4.10 Plasmids, Transfection, and Site‐Directed Mutagenesis

The coding sequences of human SRC, β‐catenin, AKT2, and VIM were cloned into the pcDNA3.1 vector (Transheep, Shanghai, China). Point mutations were introduced into wild‐type plasmids via PCR amplification and seamlessly cloned into the appropriate vector using the pEASY‐Basic Seamless Cloning and Assembly Kit (TransGen Biotech, Beijing, China) according to the manufacturer's instructions. Plasmids were transfected into KYSE150 cells using Lipofectamine 3000 (Thermo Fisher Scientific).

### 4.11 Cell Viability Assay (CCK‐8)

Cell viability was assessed using a Cell Counting Kit‐8 (CCK‐8; Dojindo, Rockville, MD, USA). Cells seeded in 96‐well plates were treated with various drug concentrations and incubated with CCK‐8 solution for 2 h at 37°C. Absorbance was measured at 450 nm to determine cell viability.

### 4.12 Colony Formation Assay

ESCC cells were plated in 6‐well plates and cultured for 2 weeks. Cells were washed with PBS, fixed with methanol for 15 min, stained with 0.1% crystal violet for 15 min, and colonies were counted for quantitative analysis.

### 4.13 Tumor Xenograft Experiment

All animal procedures were approved by the Experimental Animal Ethics Committee of Guangzhou Medical University and performed in accordance with institutional guidelines. Xenograft models were established by subcutaneous injection of 1 × 10^6^ HNM007 cells into the flanks of C57 mice. Mice were housed under standard conditions with free access to food and water. Seven days postinoculation, mice received intraperitoneal injections of cilengitide and/or anti‐PD‐1 every 3 days. The tumor length and width were measured every 3 days using calipers, and tumor volume was calculated using the formula 0.52 × length × width^2^.

### 4.14 Enzyme‐Linked Immunosorbent Assay

The concentrations of CFL1 and PAFAH1B2 in clinical serum were determined with human CFL1 ELISA kits and human PAFAH1B2 ELISA kits according to the manufacturer's instructions (Wuhan Abebio Science Co. Ltd). Briefly, serum was added and incubated for 2 h at 37°C, followed by incubation with biotin‐conjugate, streptavidin‐HRP, substrate solution, and stop solution. Absorbance was measured using a microplate reader set to 450 nm.

### 4.15 Statistical Analysis

All data processing and standard statistical analyses were conducted using R 4.5.0. Spearman's correlation analysis was employed to calculate the correlation between different modifications, with *p*‐values adjusted using the BH method. Chi‐square tests or Fisher's exact tests were used to compare categorical variables, while the Wilcoxon rank‐sum test was used for continuous variables. Survival analysis, including Cox regression and Kaplan–Meier analysis, was performed using the R packages survival (v 3.8‐3) and survminer (v 0.5.0). ROC curve calculations were conducted using the R package pROC (v 1.18.5), and time‐dependent ROC curves for prognostic signatures were generated using the R package timeROC (v 0.4). All statistical tests were two‐sided, with *p* < 0.05 considered statistically significant.

## Author Contributions

J.Y.L., S.J.L., and X.M.Y. contributed equally to this work. J.Y.L., S.J.L., and X.M.Y. designed the research studies, acquired and analyzed data, conducted the experiments, and drafted the manuscript. Q.E.M., M.W.L., Y.Z.W., Z.C.L., C.W.G., J.X.C., W.C.H., T.Y., Y.H., and X.Y.P. critically revised the manuscript for important intellectual content. M.L., Z.G.L., J.L., and B.S.Z. provided technical and/or material support. W.W.X., B.L., J.B.L., and F.Z. acquired funding, conceived and designed the study, and supervised the study. All authors have read and approved the final manuscript.

## Funding

This work was supported by the National Natural Science Foundation of China (U25A20102, 82273141, 82273368, 82573931, 82503440, and 82373259); the Chronic Disease Management Research Project of National Health Commission Capacity Building and Continuing Education Center (GWJJMB202510022191); the Outstanding Youth Program of Guangdong Natural Science Research (2023B1515020012); the Guangzhou Education Bureau Yangcheng Scholar Research Project (202235385); the Science and Technology Program of Guangzhou (2025A03J3288); the Guangzhou National Laboratory and State Key Laboratory of Respiratory Disease (GZNL2025B01003); the Guangzhou Municipal and Guangdong Provincial Key Laboratory of Protein Modification and Diseases; Henan Province Science and Technology Key Project (242102311124); and the Talent Development Foundation of The First Dongguan Affiliated Hospital of Guangdong Medical University (GCC2025006).

## Ethics Statement

All animal experiments were approved by the Ethics Committee for Animal Experiments of Guangzhou Medical University (G2023‐204), and the mice were cared for under standard conditions according to institutional guidelines. The human specimens were collected and approved by the Ethics Committee of Shanghai Chest Hospital, Shanghai Jiao Tong University (No. KS(Y)22278). Written informed consent was obtained from all participants.

## Conflicts of Interest

The authors declare no conflicts of interest.

## Supporting information




**Figure S1: Identification and annotation of ESCC key module proteins. (A)** Investigating network topology through multiple soft‐threshold power configurations. Up: Assessing the scale‐free topology fit index to soft‐threshold power. Bottom: Assessing the mean connectivity to soft‐threshold power. **(B)** Gene dendrogram with Dynamic Tree Cut modules (average linkage). **(C)** Correlation analysis between module eigengenes and clinical traits. *p*‐value levels are denoted as ns, *p* > 0.05; *, *p* < 0.05; **, *p* < 0.01; and ***, *p* < 0.001. **(D)** Heatmap showing the Khib of tumor‐specific purple module proteins across tissues. **(E)** Heatmap showing the Khib of LN‐specific brown module proteins specifically upregulated in LN. **(F)** Reactome gene sets enrichment analysis of the tumor‐specific purple module. **(G)** Reactome gene sets enrichment analysis of the LN‐specific brown module. **(H)** Disease‐free survival analysis of tumor‐specific purple module proteins. Red: risk factors (Hazard Ratio > 1, *p* < 0.05), blue: protective factors (Hazard Ratio < 1, *p* < 0.05). **(I)** Disease‐free survival analysis of LN‐specific brown module proteins. Red: risk factors (Hazard Ratio > 1, *p* < 0.05), blue: protective factors (Hazard Ratio < 1, *p* < 0.05).
**Figure S2: Validation of Khib‐phosphorylation crosstalk. (A)** The modification sites of Khib. **(B)** The modification sites of phosphorylation. **(C)** The top 10 conserved Khib motifs. **(D)** Immunoprecipitation results demonstrated crosstalk between phosphorylation at AKT2 S34 and Khib modification at K168. **(E)** Immunoprecipitation results showed that the crosstalk between phosphorylation and Khib of VIM. **(F‐G)** AKT2 **(F)** and VIM **(G)** promote ESCC cell invasion in a manner dependent on their Khib modification and phosphorylation modification. **(H‐I)** Transwell assays were performed to determine the invasive abilities of ESCC cells after overexpression of different SRC/β‐catenin mutants.
**Figure S3: The phosphorylation of tumor‐specific purple module and LN‐specific brown module in ESCC. (A)** Heatmap showing phosphorylation of LN‐specific brown module proteins elevated in LN. **(B)** GO enrichment of crosstalk protein belonging to LN‐specific brown module. **(C)** The protein level of MYH10 (P35580) were up‐regulated in LN. **(D)** The RNA level of MYH10 were up‐regulated in tumor. **(E)** MYH10 promote ESCC cell invasion in a manner dependent on their Khib modification and phosphorylation modification. **(F)** Heatmap showing phosphorylation of tumor‐specific purple module proteins. **(G)** The protein level of SMC1A (Q14683) were up‐regulated in tumor and LN. **(H)** The RNA level of SMC1A were up‐regulated in tumor or LN.
**Figure S4: Survival analysis of signature proteins and univariable Cox regression analysis. (A)** Kaplan‐Meier curves of OS according to ARHGDIA. **(B‐E)** Kaplan–Meier curves of DFS according to TPD52L2 **(B)**, HSPD1 **(C)**, HNRNPK **(D)**, and HNRNPU **(E)**. **(F‐G)** Univariable Cox regression analysis of OS **(F)** and DFS **(G)** signatures. **(H‐I)** LASSO analysis identified 10 candidate crosstalk proteins. **(J)** The protein level of CFL1 were up‐regulated in N+ samples. **(K)** The protein level of PAFAH1B2 were up‐regulated in N0 samples. **(L)** ROC analysis of SCC‐Ag for predicting LN in validation set.
**Figure S5: Functional analysis of three molecular subtypes. (A)** The CDF curves of consensus cluster for each K. **(B)** GO enrichment of upregulated proteins belonging to subtype 1. **(C)** KEGG enrichment of upregulated proteins belonging to subtype 2. **(D)** Heatmap showing the cancer hallmark‐related pathway activities across subtypes.
**Figure S6: Cilengitide enhances immunotherapy efficacy. (A)** The 50% inhibitory concentration (IC50) of cilengitide was determined in AKR cells. **(B‐C)** Cell viability assay **(B)** and colony formation assay **(C)** were performed to evaluate the effect of cilengitide on AKR cell proliferation. **(D)** The effect of cilengitide on the expression of S3 subtype signature proteins was validated by Western blotting. **(E)** Representative image of tumors from the control and drug‐treated group. **(F‐G)** Bar graphs showing tumor volume **(F)** and weight **(G)** indicate that the combination of cilengitide and anti‐PD‐1 significantly suppressed tumor growth. **(H)** Histological analysis of major organs of mice showing no significant changes among groups.
**Table S7**. REMARK Statement Checklist.

**Table S1**. Functional enrichment analysis of T and LN modules.
**Table S2**. Shared motifs between Khib and phosphorylation.
**Table S3**. Crosstalk protein list of LN module and T module.
**Table S4**. Functional enrichment analysis of crosstalk proteins of LN modules.
**Table S5**. Clinical information of serum cohort.
**Table S6**. Functional enrichment analysis of each subtype.

## Data Availability

The publicly proteomic data can be accessed from the PRIDE database under accession number: PXD021701. The RNA‐seq data generated in this study are publicly available in the Gene Expression Omnibus (GEO) at GSE264503. All raw data for the proteomic, phosphoproteomic, and Khib proteomic analyses have been uploaded to the PRIDE with the subproject ID (PXD037295, PXD051858, and PXD038252, respectively).
