# Supplementary material for: 2‐Hydroxyisobutyrylation and Phosphorylation Crosstalk Guides Metastasis Prediction and Immunotherapy in Esophageal Squamous Cell Carcinoma
Source: MedComm (2020). 2026 Jun 19;7(7):e70815. doi: 10.1002/mco2.70815 (PMC13280666; doi:10.1002/mco2.70815)
Supplement: Supplementary file 1 — Figure S1: Identification and annotation of ESCC key module proteins. (A) Investigating network topology through multiple soft‐threshold power configurations. Up: Assessing the scale‐free topology fit index to soft‐threshold power. Bottom: Assessing the mean connectivity to soft‐threshold power. (B) Gene dendrogram with Dynamic Tree Cut modules (average linkage). (C) Correlation analysis between module eigengenes and clinical traits. p‐value levels are denoted as ns, p > 0.05; *, p < 0.05; **, p < 0.01; and ***, p < 0.001. (D) Heatmap showing the Khib of tumor‐specific purple module proteins across tissues. (E) Heatmap showing the Khib of LN‐specific brown module proteins specifically upregulated in LN. (F) Reactome gene sets enrichment analysis of the tumor‐specific purple module. (G) Reactome gene sets enrichment analysis of the LN‐specific brown module. (H) Disease‐free survival analysis of tumor‐specific purple module proteins. Red: risk factors (Hazard Ratio > 1, p < 0.05), blue: protective factors (Hazard Ratio < 1, p < 0.05). (I) Disease‐free survival analysis of LN‐specific brown module proteins. Red: risk factors (Hazard Ratio > 1, p < 0.05), blue: protective factors (Hazard Ratio < 1, p < 0.05). Figure S2: Validation of Khib‐phosphorylation crosstalk. (A) The modification sites of Khib. (B) The modification sites of phosphorylation. (C) The top 10 conserved Khib motifs. (D) Immunoprecipitation results demonstrated crosstalk between phosphorylation at AKT2 S34 and Khib modification at K168. (E) Immunoprecipitation results showed that the crosstalk between phosphorylation and Khib of VIM. (F‐G) AKT2 (F) and VIM (G) promote ESCC cell invasion in a manner dependent on their Khib modification and phosphorylation modification. (H‐I) Transwell assays were performed to determine the invasive abilities of ESCC cells after overexpression of different SRC/β‐catenin mutants. Figure S3: The phosphorylation of tumor‐specific purple module and LN‐specific brown module in [file MCO2-7-e70815-s001.docx]

**2-hydroxyisobutyrylation and phosphorylation crosstalk guides metastasis prediction and immunotherapy in esophageal squamous cell carcinoma**

Junyi Li^1#^, Shujun Li^2#^, Xiaomei Yu^2#^, Qier Mu^3^, Maowen Luo^4^, Yuzhen Wang^2^, Zhichao Liu^5^, Chengwei Gu^6^, Jiaxi Chen^3^, Wangcheng He^2^, Tong Yang^2^, Yan He^3^, Xiaoya Pei^3^, Mao Lin^3, 7^, Zhigang Li^5^, Jun Liu^4^, Baosheng Zhao^6^, Fan Zhang^8*^, Jinbao Liu^2*^, Bin Li^1*^, Wenwen Xu^2*^

^1^ The Fifth Affiliated Hospital, Guangzhou Medical University, Guangzhou, China; State Key Laboratory of Metabolic Dysregulation and Prevention and Treatment of Esophageal Cancer, School of Convergence Medicine, Zhengzhou University, Zhengzhou, China.

^2^ Guangdong Provincial Key Laboratory of Protein Modification and Degradation, School of Basic Medical Sciences, The Affiliated Traditional Chinese Medicine Hospital, Guangzhou Medical University, Guangzhou, China.

^3^ The Fifth Affiliated Hospital, Guangzhou Medical University, Guangzhou, China.

^4^ State Key Laboratory of Respiratory Disease and National Clinical Research Center for Respiratory Disease, Department of Thoracic Surgery and Oncology, the First Affiliated Hospital of Guangzhou Medical University, Guangzhou, China.

^5^ Department of Thoracic Surgery, Shanghai Chest Hospital, Shanghai Jiao Tong University School of Medicine, Shanghai, China.

^6^ Department of Thoracic Surgery, The First Affiliated Hospital of Henan Medical University. Xinxiang, China.

^7^ Dongguan Key Laboratory of Precision Medicine, Precision Medicine Center, The First Dongguan Affiliated Hospital, Guangdong Medical University, Guangdong, China.

^8^ Department of Gastrointestinal Surgery, The Fifth Affiliated Hospital of Guangzhou Medical University, Guangzhou, China.

^#^Contributed equally

^*^Correspondence:

Email: xuwen6966@163.com (Wenwen Xu)

Email: lib2128@163.com (Bin Li)

Email: jliu@gzhmu.edu.cn (Jinbao Liu)

Email: 875086089@qq.com (Fan Zhang)


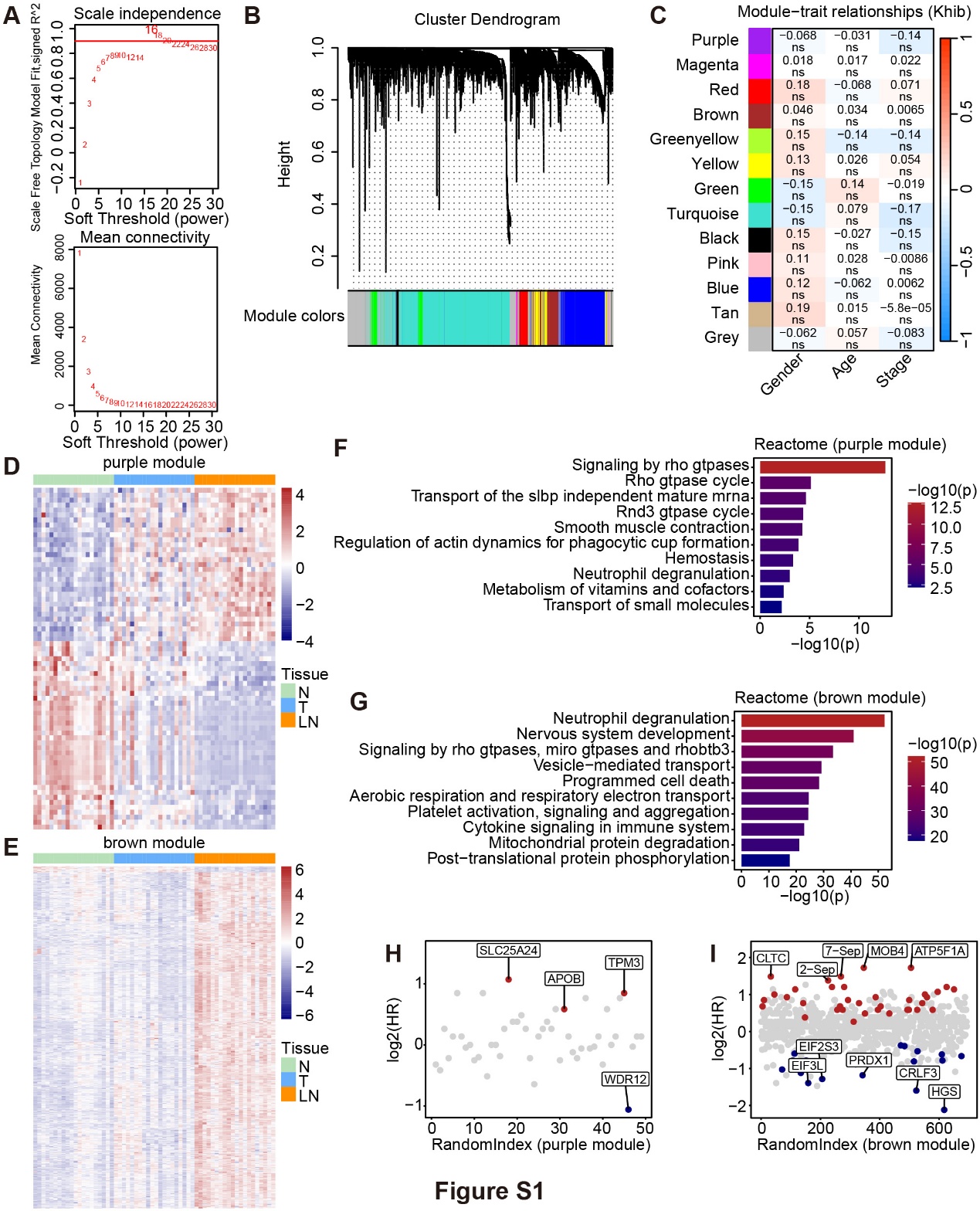


**Figure S1: Identification and annotation of ESCC key module proteins. (A)** Investigating network topology through multiple soft-threshold power configurations. Up: Assessing the scale-free topology fit index to soft-threshold power. Bottom: Assessing the mean connectivity to soft-threshold power. **(B)** Gene dendrogram with Dynamic Tree Cut modules (average linkage). **(C)** Correlation analysis between module eigengenes and clinical traits. P value levels are denoted as ns, *P* > 0.05; *, *P* < 0.05; **, *P* < 0.01; and ***, *P* < 0.001. **(D)** Heatmap showing the Khib of tumor-specific purple module proteins across tissues. **(E)** Heatmap showing the Khib of LN-specific brown module proteins specifically upregulated in LN. **(F)** Reactome gene sets enrichment analysis of the tumor-specific purple module. **(G)** Reactome gene sets enrichment analysis of the LN-specific brown module. **(H)** Disease-free survival analysis of tumor-specific purple module proteins. Red: risk factors (Hazard Ratio > 1, *P* < 0.05), blue: protective factors (Hazard Ratio < 1, *P* < 0.05). **(I)** Disease-free survival analysis of LN-specific brown module proteins. Red: risk factors (Hazard Ratio > 1, *P* < 0.05), blue: protective factors (Hazard Ratio < 1, *P* < 0.05).


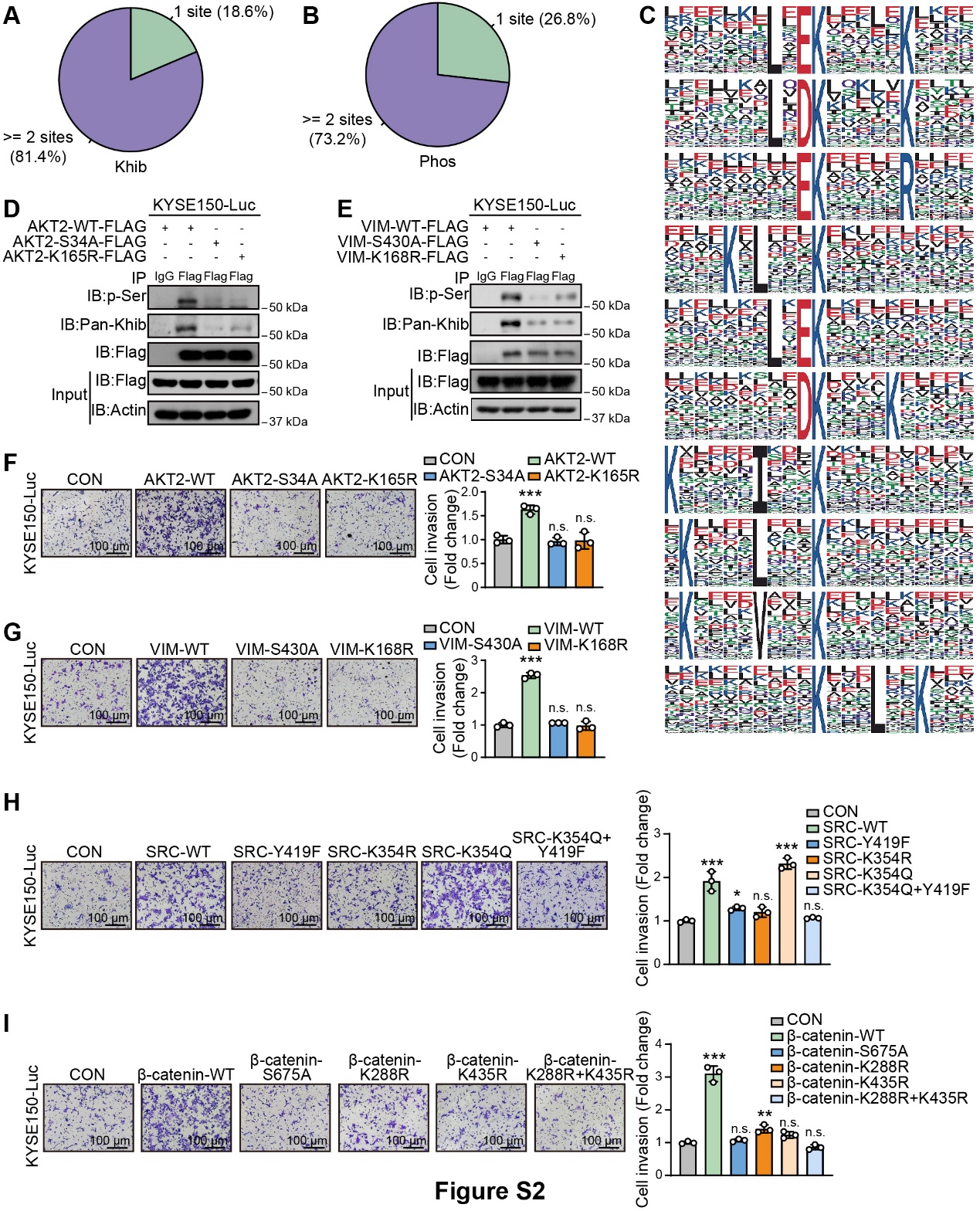


**Figure S2: Validation of Khib-phosphorylation crosstalk. (A)** The modification sites of Khib. **(B)** The modification sites of phosphorylation. **(C)** The top 10 conserved Khib motifs. **(D)** Immunoprecipitation results demonstrated crosstalk between phosphorylation at AKT2 S34 and Khib modification at K168. **(E)** Immunoprecipitation results showed that the crosstalk between phosphorylation and Khib of VIM. **(F-G)** AKT2 **(F)** and VIM **(G)** promote ESCC cell invasion in a manner dependent on their Khib modification and phosphorylation modification. **(H-I)** Transwell assays were performed to determine the invasive abilities of ESCC cells after overexpression of different SRC/β-catenin mutants.


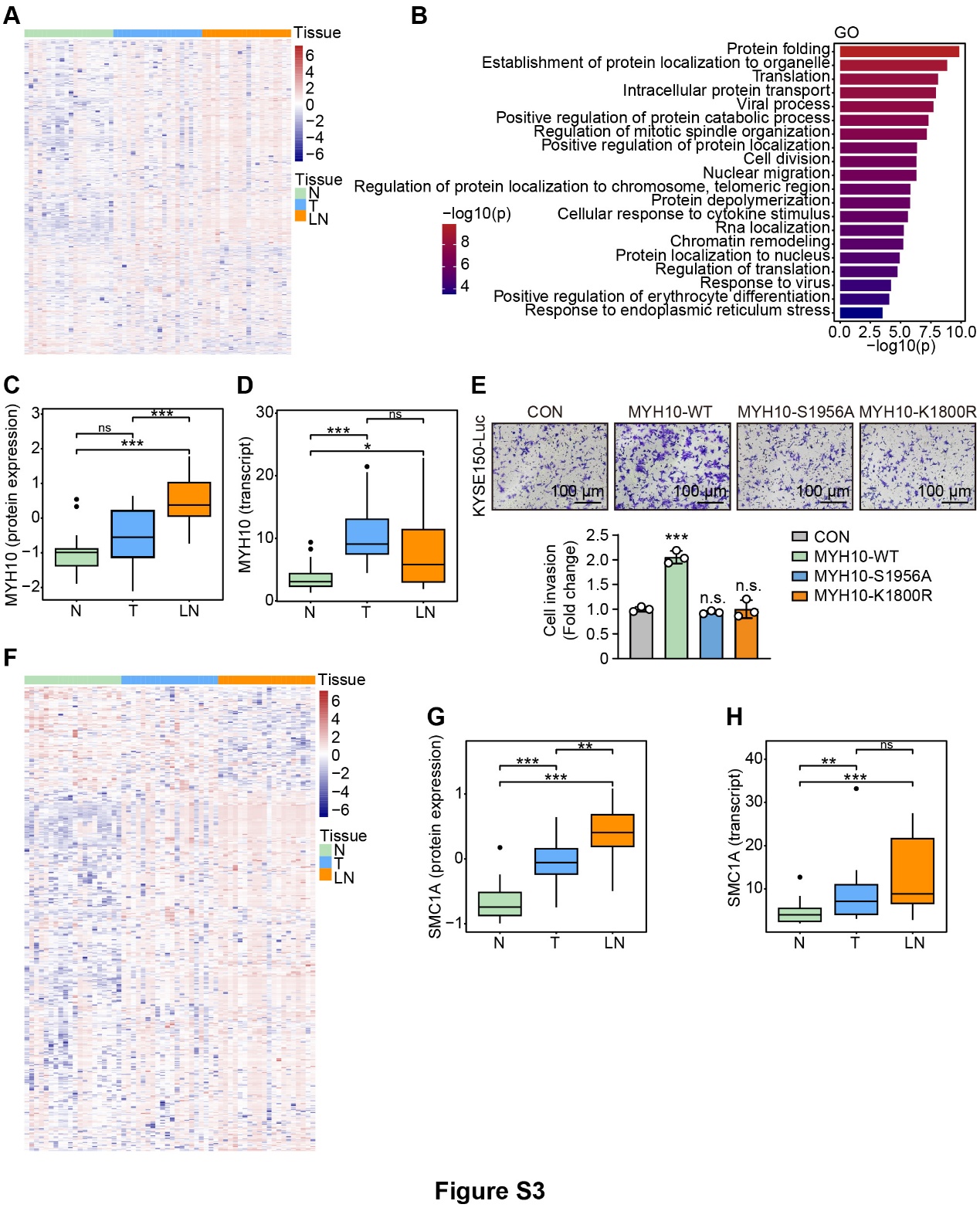


**Figure S3: The phosphorylation of tumor-specific purple module and LN-specific brown module in ESCC. (A)** Heatmap showing phosphorylation of LN-specific brown module proteins elevated in LN. **(B)** GO enrichment of crosstalk protein belonging to LN-specific brown module. **(C)** The protein level of MYH10 (P35580) were up-regulated in LN. **(D)** The RNA level of MYH10 were up-regulated in tumor. **(E)** MYH10 promote ESCC cell invasion in a manner dependent on their Khib modification and phosphorylation modification. **(F)** Heatmap showing phosphorylation of tumor-specific purple module proteins. **(G)** The protein level of SMC1A (Q14683) were up-regulated in tumor and LN. **(H)** The RNA level of SMC1A were up-regulated in tumor or LN.


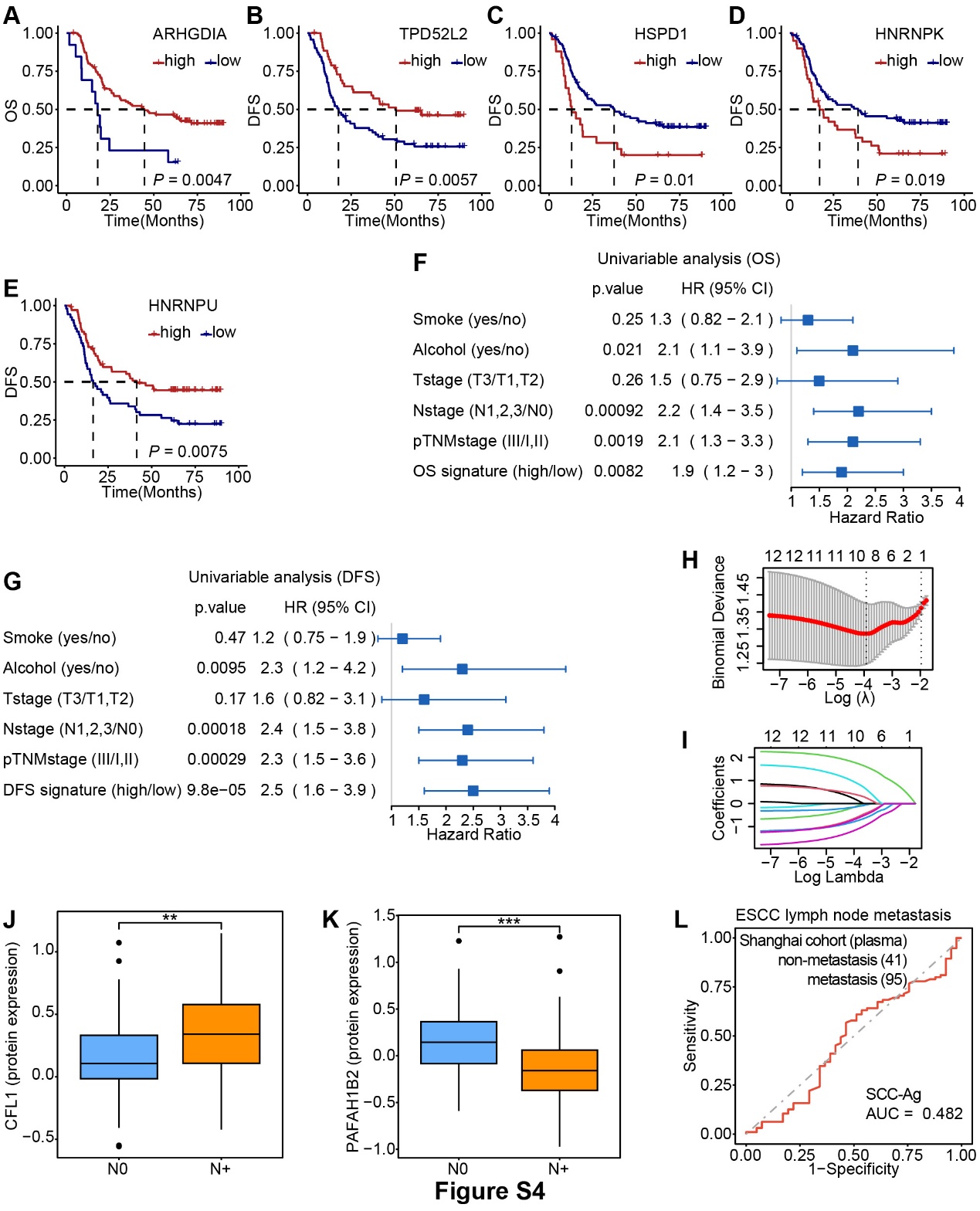


**Figure S4: Survival analysis of signature proteins and univariable Cox regression analysis. (A)** Kaplan-Meier curves of OS according to ARHGDIA. **(B-E)** Kaplan–Meier curves of DFS according to TPD52L2 **(B)**, HSPD1 **(C)**, HNRNPK **(D)**, and HNRNPU **(E)**. **(F-G)** Univariable Cox regression analysis of OS **(F)** and DFS **(G)** signatures. **(H-I)** LASSO analysis identified 10 candidate crosstalk proteins. **(J)** The protein level of CFL1 were up-regulated in N+ samples. **(K)** The protein level of PAFAH1B2 were up-regulated in N0 samples. **(L)** ROC analysis of SCC-Ag for predicting LN in validation set.


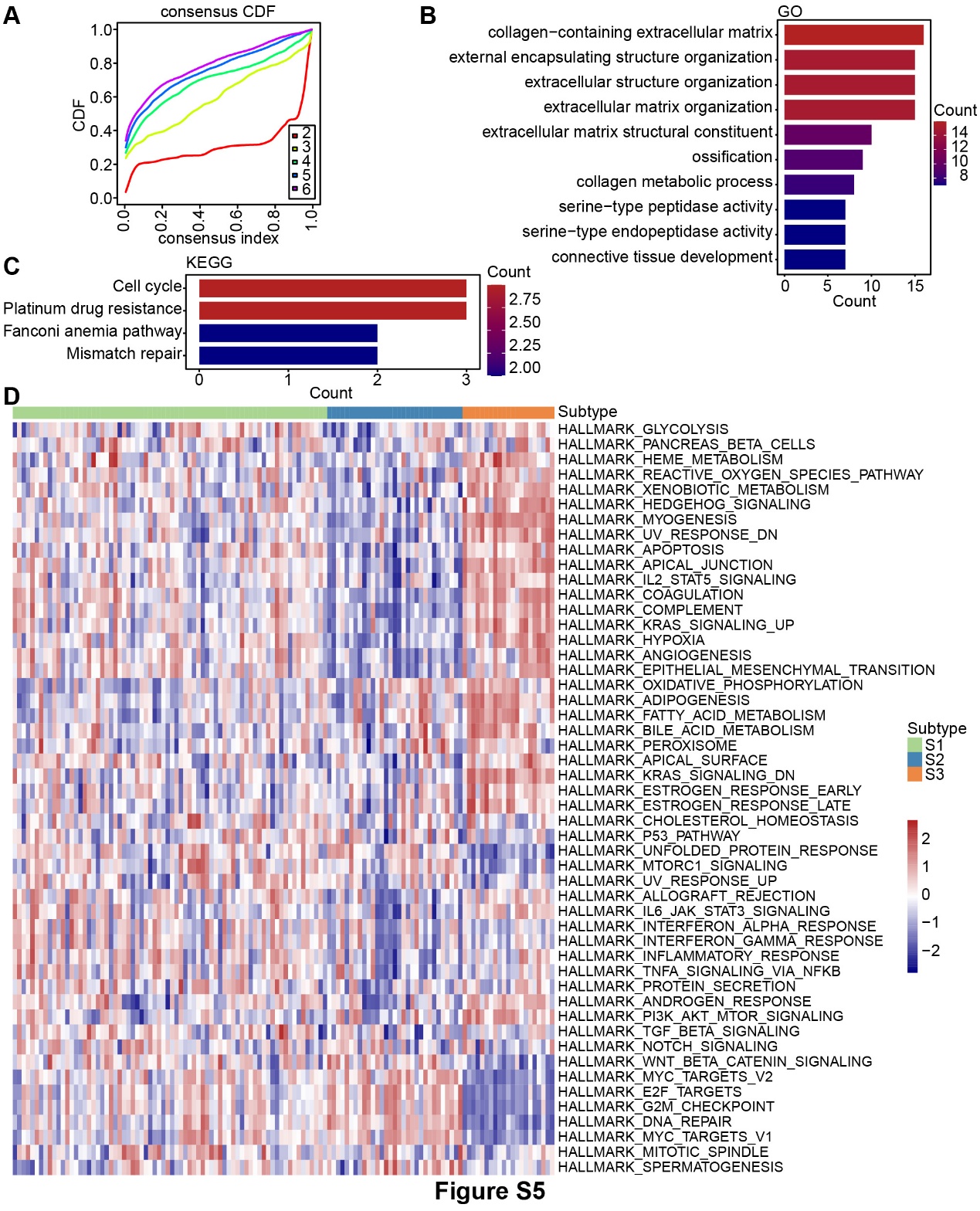


**Figure S5: Functional analysis of three molecular subtypes. (A)** The CDF curves of consensus cluster for each K. **(B)** GO enrichment of upregulated proteins belonging to subtype 1. **(C)** KEGG enrichment of upregulated proteins belonging to subtype 2. **(D)** Heatmap showing the cancer hallmark-related pathway activities across subtypes.


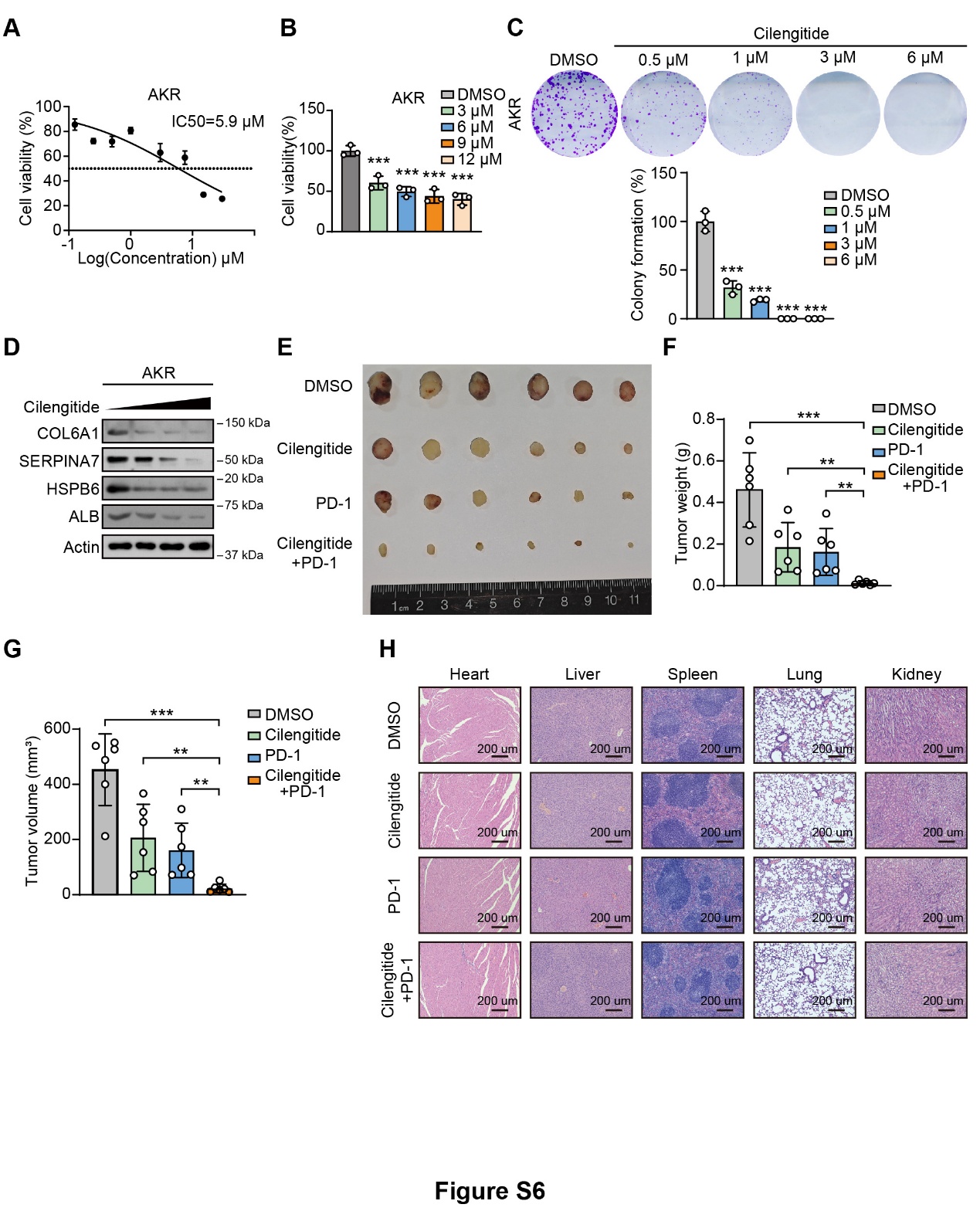


**Figure S6: Cilengitide enhances immunotherapy efficacy. (A)** The 50% inhibitory concentration (IC50) of cilengitide was determined in AKR cells. **(B-C)** Cell viability assay **(B)** and colony formation assay **(C)** were performed to evaluate the effect of cilengitide on AKR cell proliferation. **(D)** The effect of cilengitide on the expression of S3 subtype signature proteins was validated by Western blotting. **(E)** Representative image of tumors from the control and drug-treated group. **(F-G)** Bar graphs showing tumor volume **(F)** and weight **(G)** indicate that the combination of cilengitide and anti-PD-1 significantly suppressed tumor growth. **(H)** Histological analysis of major organs of mice showing no significant changes among groups.

Table S7. REMARK Statement Checklist

| **Item to be reported** | | **Page no.** |
| --- | --- | --- |
| **INTRODUCTION** | |  |
| 1 | State the marker examined, the study objectives, and any pre-specified hypotheses. | 4-5 |
| **MATERIALS AND METHODS** | |  |
| *Patients* | |  |
| 2 | Describe the characteristics (e.g., disease stage or co-morbidities) of the study patients, including their source and inclusion and exclusion criteria. | 19, 28-29 |
| 3 | Describe treatments received and how chosen (e.g., randomized or rule-based). | 19, 28-29 |
| *Specimen characteristics* | |  |
| 4 | Describe type of biological material used (including control samples) and methods of preservation and storage. | 24-27 |
| *Assay methods* | |  |
| 5 | Specify the assay method used and provide (or reference) a detailed protocol, including specific reagents or kits used, quality control procedures, reproducibility assessments, quantitation methods, and scoring and reporting protocols. Specify whether and how assays were performed blinded to the study endpoint. | 24-27 |
| *Study design* | |  |
| 6 | State the method of case selection, including whether prospective or retrospective and whether stratification or matching (e.g., by stage of disease or age) was used. Specify the time period from which cases were taken, the end of the follow-up period, and the median follow-up time. | 19, 28-29 |
| 7 | Precisely define all clinical endpoints examined. | 19, 28-29 |
| 8 | List all candidate variables initially examined or considered for inclusion in models. | 19, 28-29 |
| 9 | Give rationale for sample size; if the study was designed to detect a specified effect size, give the target power and effect size. | 19, 28-29 |
| *Statistical analysis methods* | |  |
| 10 | Specify all statistical methods, including details of any variable selection procedures and other model-building issues, how model assumptions were verified, and how missing data were handled. | 27 |
| 11 | Clarify how marker values were handled in the analyses; if relevant, describe methods used for cutpoint determination. | 27 |
| **RESULTS** | |  |
| *Data* | |  |
| 12 | Describe the flow of patients through the study, including the number of patients included in each stage of the analysis (a diagram may be helpful) and reasons for dropout. Specifically, both overall and for each subgroup extensively examined report the numbers of patients and the number of events. | 11-12 |
| 13 | Report distributions of basic demographic characteristics (at least age and sex), standard (disease-specific) prognostic variables, and tumor marker, including numbers of missing values. | 11-12 |
| *Analysis and presentation* | |  |
| 14 | Show the relation of the marker to standard prognostic variables. | 11-12 |
| 15 | Present univariable analyses showing the relation between the marker and outcome, with the estimated effect (e.g., hazard ratio and survival probability). Preferably provide similar analyses for all other variables being analyzed. For the effect of a tumor marker on a time-to-event outcome, a Kaplan-Meier plot is recommended. | 11-12 |
| 16 | For key multivariable analyses, report estimated effects (e.g., hazard ratio) with confidence intervals for the marker and, at least for the final model, all other variables in the model. | 11-12 |
| 17 | Among reported results, provide estimated effects with confidence intervals from an analysis in which the marker and standard prognostic variables are included, regardless of their statistical significance. | 11-12 |
| 18 | If done, report results of further investigations, such as checking assumptions, sensitivity analyses, and internal validation. | 11-12 |
| **DISCUSSION** | |  |
| 19 | Interpret the results in the context of the pre-specified hypotheses and other relevant studies; include a discussion of limitations of the study. | 15-19 |
| 20 | Discuss implications for future research and clinical value. | 18 |

Source: McShane LM, Altman DG, Sauerbrei W, Taube SE, Gion M, Clark GM: Reporting recommendations for tumor marker prognostic studies (REMARK). J Natl Cancer Inst 2005; 97: 1180-1184.
